# The prevalence and concentration of *Salmonella enterica* in poultry litter in the southern United States

**DOI:** 10.1371/journal.pone.0268231

**Published:** 2022-05-26

**Authors:** Laurel L. Dunn, Vijendra Sharma, Travis K. Chapin, Loretta M. Friedrich, Colleen C. Larson, Camila Rodrigues, Michele Jay-Russell, Keith R. Schneider, Michelle D. Danyluk

**Affiliations:** 1 Department of Food Science and Technology, University of Georgia, Athens, GA, United States of America; 2 Food Science and Human Nutrition Department, Citrus Research and Education Center, University of Florida, Lake Alfred, FL, United States of America; 3 Okeechobee County Cooperative Extension Service, Institute of Food and Human Nutrition University of Florida, Okeechobee, FL, United States of America; 4 Western Center for Food Safety, University of California, Davis, Davis, CA, United States of America; 5 Food Science and Human Nutrition Department, University of Florida, Gainesville, FL, United States of America; Oregon State University, UNITED STATES

## Abstract

Poultry litter is applied to crop production land in the southern United States as a waste management strategy as it is a nitrogen-rich fertilizer and plentiful throughout the region. While litter is a known reservoir for human enteric pathogens including *Salmonella enterica*, little is known regarding pathogen prevalence, concentration, and common serotypes within the material. Litter from thirteen farms across four southern states was examined for *Salmonella*. Samples (n = 490) from six of the thirteen (46.2%) farms tested positive. Thirty-three samples out of 490 (6.7%) were *Salmonella* positive. *Salmonella* was ca. 95% less likely to be collected from stacked litter piles than from the poultry house floor or pasture, and every day increase in litter age reduced the likelihood of recovering *Salmonella* by 5.1%. When present, concentrations of *Salmonella* in contaminated poultry litter were variable, ranging from <0.45 to >280,000 MPN/g. The most prevalent serotypes found were Kentucky (45.5%), Kiambu (18.2%), and Michigan (12.1%). *Salmonella* Kentucky also had the greatest distribution and was found on 4 of the 6 (66.7%) positive farms. Results from this survey demonstrated that *Salmonella* prevalence and concentration in poultry litter is highly variable, and good agricultural practices are critical to safely use poultry litter as a soil amendment on fresh produce fields.

## Introduction

Poultry litter contains waste material from broiler, breeder, and layer operations, and is comprised of bedding, feed, feathers, manure, and mortalities accumulated throughout production. Physical, chemical, and microbial quality of litter often varies depending on rearing method [1, 2]. Litter and other animal manure-containing material can harbor human enteric pathogens including *Salmonella enterica* subspecies *enterica* [3], *Campylobacter jejuni* [4], and Shiga-toxigenic *Escherichia coli* [5, 6], which, if not properly treated, can contaminate produce when applied to agricultural land [7, 8]. Microbial population concentration and diversity in litter can be affected by storage time, storage location, temperature, moisture, and other factors [9].

Composting and physical dry-heating are the most commonly used treatment methods to reduce potential foodborne pathogens in poultry litter [10]. The use of untreated biological soil amendments of animal origin (BSAAO) is considered a high-risk agricultural practice due to the increased likelihood of enteric pathogen contamination and subsequent spread to produce. Untreated BSAAO include materials such as raw or stacked manure (including poultry waste) that have not undergone treatment (e.g., composting) to reduce pathogens [10]. However, it is common practice on many mixed crop-livestock farms or produce farms near livestock operations to land apply untreated BSAAO as they are readily available and generally less expensive than commercial fertilizers [11, 12]. Land application is also a crucial means of waste management in areas where litter supply exceeds demand for fertilizer or ruminant feed [11]. However, over-application is an environmental concern as excessive phosphorous and nitrogen levels can result in eutrophication of water sources [13–15]. With United States (US) agriculture generating an estimated 335 million tons of dry matter waste annually, of which poultry production accounts for 10.2 million tons, litter may either be considered an invaluable resource or a waste management dilemma depending on regional supply and demand [12, 16].

The use of soil amendments on many produce commodities is regulated by the Food and Drug Administration (FDA) through the Standards for the Growing, Harvesting, Packing, and Holding of Produce for Human Consumption, otherwise called the Produce Safety Rule (PSR; [17]). There is currently no regulatory standard on application-to-harvest interval for untreated BSAAO when used on fresh produce. The FDA has listed this portion of the rule as “reserved” pending further investigation [17] but recommends that produce growers adhere to the National Organic Program standard application-to-harvest interval of 120 days for produce likely to contact the ground when untreated BSAAO is present and 90 days for produce that is unlikely to contact the ground and applied, untreated BSAAO [18]. If this 90/120-day interval is improbable and produce contact is likely, a BSAAO should be composted or otherwise treated [19]. More research is necessary to determine the prevalence and concentration of foodborne pathogens in BSAAO, including untreated poultry litter, to aid in the establishment of ideal application-to-harvest intervals that will effectively reduce the risk of crop contamination.

The objective of this research was to determine the prevalence, diversity, and concentration of *Salmonella* in poultry litter obtained through convenience sampling from farms across the southeast US. Material sampled included litter from large-scale broiler houses, some large-scale breeder houses, and ground samples from small- and medium-sized pasture raised broilers. While the number and scale of operations sampled may not be directly proportional to the number of poultry houses found throughout the region, it is representative of the many types of operations found within the Southeast. The findings are part of a multi-regional study to inform FDA’s risk assessment by providing key information regarding contributors that may affect the microbiological quality of poultry litter used as a BSAAO on fresh produce.

## Materials and methods

### Farm enlistment and sample collection

Collection of poultry litter from farms occurred from August 2017 to May 2018. Participating farms were identified with the assistance of Cooperative Extension, local poultry associations, or during produce safety-related meetings. Thirteen farms located in Alabama (n = 7) Florida (n = 2), Georgia (n = 2), and Texas (n = 2) volunteered to provide samples during the first round of sampling. Farms were visited twice, with three exceptions. During the second round of sampling, one Georgia cotton farm that previously supplied locally purchased litter had none available and was therefore unable to provide a sample. A duplicate sample was also not collected from a breeder farm in Alabama because all remaining litter was sold immediately after the first round of sampling. A flock in Texas was sold and the pasture repurposed before a second sampling could occur.

At each operation, three piles were sampled with seven samples collected by hand from each pile (n = 21); gloves were changed between each sample. Two exterior samples weighing no less than 30 g were collected from the pile surface and placed into sterile Whirl-Pak® bags (Whirl-Pak, Madison, WI). Five interior 30 g samples were collected at different depths from holes dug (using an ethanol-cleaned shovel) 0.5 to 3 ft into the piles. When litter was not stacked (i.e., on the chicken house floor or pasture), a manure sample was collected directly from the ground. Samples were immediately placed on ice and transported to the lab for processing within 12 h, except for the first sampling at Farm 5 when an ice storm delayed return flights from Texas. These samples were kept iced in coolers for 48 h prior to processing. During sample processing from the first visit to Farm 1, a hurricane disrupted power at the laboratory. Enrichment reservoirs from these samples were stored on ice for 4 d until power was restored, and processing was completed.

### Presence/Absence test for *Salmonella*

**Fig 1:**
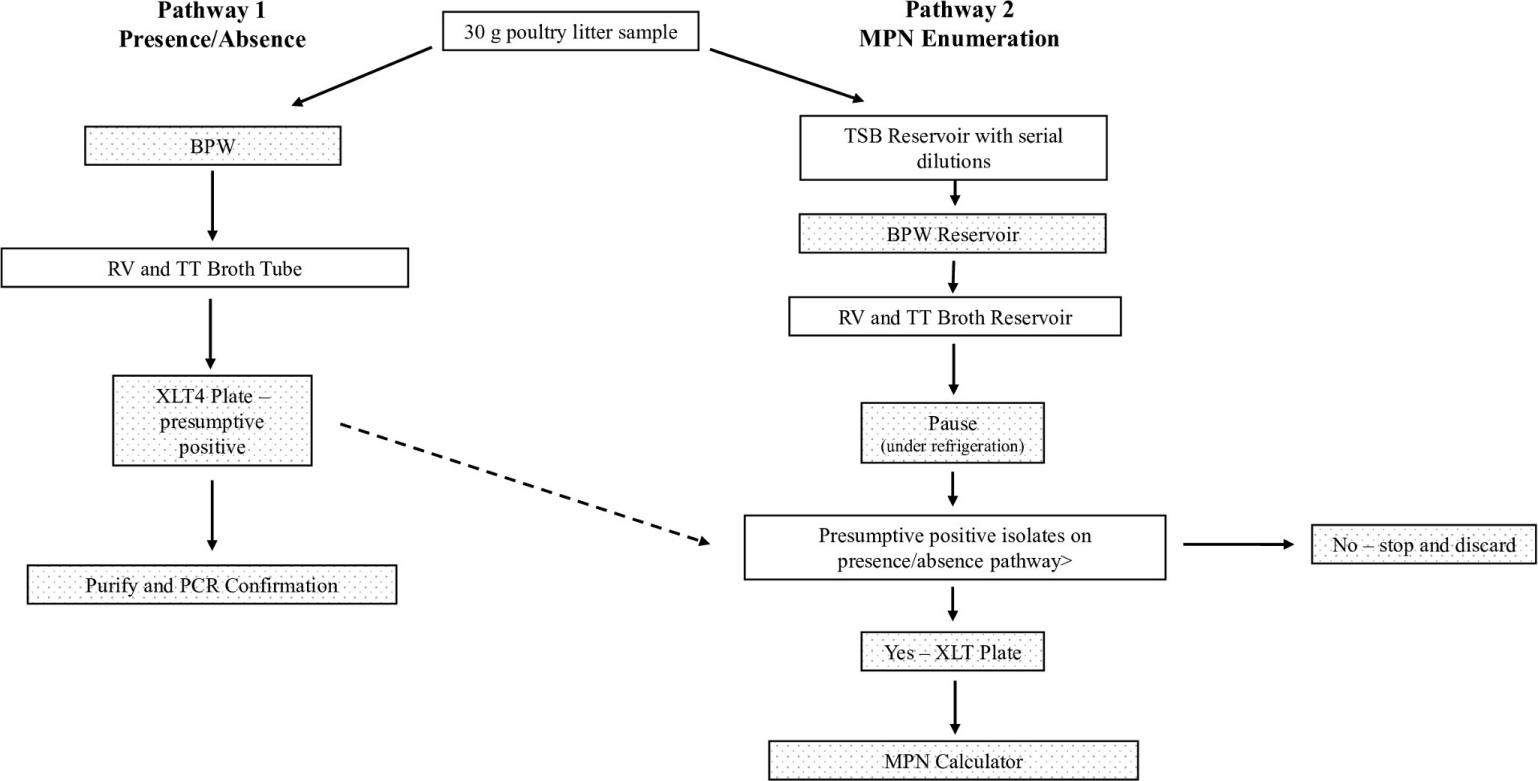
Workflow diagram for *Salmonella enterica* presence/absence method and most probable number enumeration from 30 g poultry litter samples collected from southern U.S. farms.

Thirty grams of litter from each sample were weighed out and combined with 270 mL of buffered peptone water (BPW; BD, Franklin Lakes, NJ) in a sterile Whirl-Pak® bag (Fig 1); sample weights were not adjusted according to moisture content. Each bag was vigorously hand-shaken/massaged for one min. Bags were transferred to a shaking incubator set to 37°C and 50 rpm. After 24 h incubation, 1 mL was transferred from the Whirl-Pak® bag (without filter) into 9 mL of BPW, which was returned to the shaking incubator for an additional 20 h at 37°C and 50 RPM. After 20 h, 100 μL was transferred into tubes containing 10 mL Rappaport-Vassiliadis (RV; BD, Franklin Lakes, NJ) broth and 1000 μL was transferred into 10 mL Tetrathionate (TT; BD, Franklin Lakes, NJ) broth. Tubes were incubated at 42°C for 48 h (RV) or 24 h (TT). After incubation, 10 μL broth was streaked onto a Xylose Lysine Tergitol-4 (XLT-4; BD, Franklin Lakes, NJ) plate and incubated at 37°C for 24–48 h. Up to four presumptive positive colonies (black, or red with a black center) were re-streaked onto individual XLT-4 plates and incubated an additional 24–48 h prior to PCR confirmation.

### *Salmonella* quantification

A modified most probable number (MPN) method was used for the enumeration of *Salmonella* from litter [5, 20, 21] The same 30 g litter sample (combined with 270 mL BPW in a Whirl-Pak® bag) used for presence/absence was also used for MPN quantification. Immediately after mixing litter with BPW, 5.5 mL was pipetted from the Whirl-Pak® bag into the first row of a 48-well, deep well reservoir; this was replicated four times for each sample. The sample was serial diluted by removing 500 μL from row one and adding it to row two, which contained 4.5 mL tryptic soy broth (TSB; BD, Franklin Lakes, NJ). The contents of row two were mixed by pipetting and expelling the volume within the well several times. The pipet tip was replaced, 500 μL was removed from row two, added to row three, and the process was repeated until a final dilution of 10^−6^ was achieved. Reservoirs were sealed using breathable adhesive film and incubated in a shaking incubator at 37°C and 50 rpm for 20 h. After incubation, 500 μL from each well was transferred into the corresponding well in a new reservoir; each well contained 4.5 mL BPW. Reservoirs were re-sealed with breathable adhesive film and incubated for 20 h at 37°C and 50 RPM. The following day, 50 μL and 500 μL were transferred from each well of the BPW reservoir into the corresponding well in reservoirs containing either 5 mL RV or TT broth, respectively. RV reservoirs were incubated for 48 h and TT incubated for 24 h, both at 42°C. Reservoirs were removed from incubation and stored at refrigeration for four days until the results of the presence/absence test determined which positive samples would continue for further processing. A validation study conducted by Marik et al. [21] determined that refrigerated storage for this duration did not significantly impact *Salmonella* recovery.

When positive samples were identified in the presence/absence test above, the corresponding RV and TT reservoirs were removed from refrigeration. Using a sterile inoculating needle, each well was streaked onto individual XLT-4 agar plates and incubated at 37°C for up to 48 h. Presumptive positive colonies were re-streaked onto fresh XLT-4 agar, incubated for 24–48 h and PCR confirmed. MPNs were calculated based on corresponding dilution wells per sample. Positive isolates were submitted to the National Veterinary Services Laboratory (Ames, Iowa 50010) for serotyping.

### PCR confirmation

Sample DNA was extracted by combining a loopful of culture from an XLT-4 plate with 100 μL DNase free water in a 1.5 mL microcentrifuge tube. Sample tube was boiled for 20 min at 100°C, centrifuged for 10 min at maximum speed (10,000 ✕ g), after which the supernatant was removed and reserved in a sterile 1.2 mL microcentrifuge tube. The presence of *invA* was used to detect *Salmonella* using the forward 5’ GTC ACG GAA GAA GAG AAA TCC GTA CG 3’ and reverse 5’ GGG AGT CCA GGT TGA CGG AAA ATT T 3’ primers [22]. *Salmonella* Newport (MDD313, tomato outbreak isolate, University of Florida culture collection) was used as a positive control. The PCR reagent concentrations for each 50 μL reaction were: 19 μL double distilled water, 25 μL DreamTaq Green Master Mix (Thermo Fisher Scientific, Waltham, MA), 2 μL of 10 μM forward primer, 2 μL of 10 μM reverse primer, and 2 μL of DNA template. PCR conditions included initial denaturation which occurred at 95°C for 3 min, followed by 35 cycles of denaturation at 95°C for 30 sec, annealing at 53.9°C for 30 sec, and extension at 72°C for 1 min. Final extension occurred at 72°C for 5 min, then samples were held at 4°C until removal from thermocycler. Gel electrophoresis was run on 2.0% agarose gels in 1× TBE buffer (Bio-Rad, Hercules, CA, USA) at 120 V for 60 min. Gels were stained by submerging in 400 mL ethidium bromide (40 mg/mL) for 20 min, then destained twice in 400 mL deionized water for 20 min. The MultiDoc-It Digital Imaging System (UVP, Upland, CA) was used for UV visualization.

### Statistical analysis

A logistic regression analysis for the prevalence of *Salmonella* was performed using the software RStudio version 3.3.2 (R Foundation for Statistical Computing, Vienna, Austria). The analysis used pile temperature, location of collected sample (e.g., surface of the pile, interior of the pile, or ground), whether litter was stored under protection (e.g., protected or not protected), and pile age (e.g., days since last bird access) to indicate the likelihood of *Salmonella* to be present on samples. The logistic regression model’s performance was validated based on the McFadden (pseudo R2) values between 0.2–0.4 and accuracy test of the logistic regression analysis.

## Results

Four hundred and ninety samples were collected from 70 piles (dry stacks) or the ground (poultry houses and pastures) from 13 farms across four southern states. In general, *Salmonella* was isolated from 33 (6.7%) of the total 490 samples, in which nearly half of positive samples were found on farms located in Georgia (54.5%, 18/33), followed by Texas (30.3%, 10/33), Alabama (12.1%, 4/33), and Florida (3.0%, 1/33; Table 1). Among samples collected from 70 piles, *Salmonella* was detected in 14 piles (20%), in which half of the positive piles were from farms in Georgia (50%, 7/14; Table 1).

**Table 1:** Number and concentration of *Salmonella*-positive piles and samples from each farm sampling. Sample concentration for positive samples reported in most probable number per gram (MPN/g).

Farm	State	Visit*	Month	Number of positive piles	Number of positive samples	Concentration Range (MPN/g)
1	GA	1	Aug	3/3	11/21 (52.4%)	<0.45–460
		2	Mar	2/3	2/21 (9.5%)	>280000
2	GA*	1	Oct	2/3	5/21 (23.8%)	>280000
		2	.	.	.	.
3	FL	1	Jan	0/3	0/21	.
		2	Apr	0/3	0/21	.
4	FL	1	Mar	1/3	1/21 (4.8%)	<0.45
		2	May	0/3	0/21	.
5	TX	1	Jan	3/3	10/21 (47.6%)	<0.45 - >280000
		2	May	0/3	0/21	.
6	TX*	1	.	.	.	.
		2	May	0/3	0/21	.
7	AL	1	Nov	0/3	0/21	.
		2	Feb	0/3	0/21	.
8	AL	1	Dec	1/3	1/21 (4.8%)	>280000
		2	Mar	0/3	0/21	.
9	AL	1	Dec	0/3	0/21	.
		2	Mar	0/3	0/21	.
10	AL	1	Feb	0/3	0/21	.
		2	Mar	0/3	0/21	.
11	AL	1	Feb	0/3	0/21	.
		2	Mar	0/3	0/21	.
12	AL	1	Feb	0/3	0/21	.
		2	May	0/3	0/21	.
13	AL*	1	Feb	2/4	3/28 (10.7%)	<0.45 - >280000
		2	.	.	.	.
**Total**	** **	** **	** **	**14/70**	**33/490 (6.7%)**	**<0.45 - >280000**

* Farm only sampled once.

Litter from 6 of the 13 (46.2%) farms was positive for *Salmonella* with concentrations ranging from <0.45 to >280,000 MPN/g (Table 1). Positive samples came from both farms in Georgia (100%, 2/2), one farm in Florida (50%, 1/2), one farm in Texas (50%, 1/2), and two farms in Alabama (28.6%, 2/7; Table 1). Only one farm, located in Georgia, was positive for *Salmonella* on both visits. On all other *Salmonella*-positive farms, the pathogen was only isolated on one of the visits (Table 1).

One hundred and sixty-five ground samples were collected from poultry house floor or pasture, in which *Salmonella* was isolated from 23 of the total samples (13.9%, 23/165); while *Salmonella* was detected in 10 samples from a total of 325 samples collected directly from piles of stacked litter (3.1%, 10/325). As shown in Table 2, ground samples accounted for 69.7% (23/33) of total positive samples. The remaining positives were collected from the piles, with interior samples accounting for 24.2% (8/33) of positives, while samples collected from the pile exterior contributed to 6.1% (2/33; Table 2) of the total positives collected. Forty-eight percent of positive samples (16/33) were collected from fresh litter (day one), while 36.4% (12/33) and 15.1% (5/33) were collected from litter as old as 7 and 21 days, respectively (age since last bird access; Table 2).

**Table 2:** Concentration of *Salmonella* in positive samples collected from piles, houses, or pastures in the southern United States.

Farm	Visit	Sample Location	Sample age (days)	*Salmonella* concentration (MPN/g)
1	1	House Floor	1	8.2
	1	House Floor	1	460
	1	House Floor	1	<0.45
	1	House Floor	1	<0.45
	1	House Floor	1	120
	1	House Floor	1	<0.45
	1	House Floor	1	<0.45
	1	House Floor	1	<0.45
	1	House Floor	1	<0.45
	1	House Floor	1	<0.45
	1	House Floor	1	<0.45
	2	House Floor	1	>280000
	2	House Floor	1	>280000
2	1	Interior	21	>280000
	1	Interior	21	>280000
	1	Interior	21	>280000
	1	Interior	21	>280000
	1	Exterior	21	44000
4	1	Interior	1	<0.45
5	1	Pasture	7	>280000
	1	Pasture	7	4600
	1	Pasture	7	<0.45
	1	Pasture	7	320
	1	Pasture	7	1100
	1	Pasture	7	12000
	1	Pasture	7	19
	1	Pasture	7	4.6
	1	Pasture	7	1.6
	1	Pasture	7	8.2
8	1	Exterior	7	>280000
13	1	Interior	1	48000
	1	Interior	1	280000
	1	Interior	1	<0.45

Individual logistic analysis of the variables in litter samples showed that *Salmonella* was 96% and 95% less likely to be isolated from the exterior and interior of the pile, respectively, when compared to the ground samples collected from poultry houses or pastures (Exterior: OR = 0.13, 95% CI = 0.02–0.59; Interior: OR = 0.16, 95% CI = 0.05–0.44; Table 3). Also, for every day increase in pile age, the likelihood of isolating *Salmonella* from the litter was reduced by 5% (OR = 0.96, 95% CI = 0.92–0.99; Table 3). Sample temperature and whether the pile was stored under protection were not statistically significant in the logistic regression analysis.

**Table 3:** Individual logistic analysis of the variables in poultry litter samples collected from farms in the southeastern United States.

Variables		Coefficient	OR	95% CI	*P-*value
Sample Temp (°C)		0.06	1.06	0.99–1.15	0.119
Location	Exterior of pile	-3.00	0.05	0.004–0.34	0.009**
	Interior of pile	-2.80	0.06	0.002–0.3	0.003**
Protection	None	2.19	8.91	0.67–402.2	0.170
Sample age		-0.05	0.95	0.89–0.98	0.032*

Logistic regression analysis was performed to predict the likelihood of *Salmonella* to be isolated from poultry litter samples.

* *p* < 0.5

** *p* < 0.01

From the 33 isolates collected, the most frequently found *Salmonella* serotypes included Kentucky (45.5%, 15/33), Kiambu (18.2%, 6/33), and Michigan (12.1%, 4/33). Other serovars comprising 6% or fewer positive samples included Anatum (6.1%, 2/33), Braenderup (6.1%, 2/33), Newport (3.0%, 1/33), Seftenberg (3.0%, 1/33), Mbandaka (3.0%, 1/33), and Saintpaul (3.0%, 1/33).

## Discussion

Nearly 7% of poultry litter samples (33/490) from 13 southern farms were positive for *Salmonella* (Table 1). When isolated from litter, *Salmonella* concentrations were variable and ranged from < 0.45 MPN/g to >280,000 MPN/g; two farms had at least 1 positive sample below and 1 positive sample above the limits of quantification (Table 1). Even though piled litter had a lower prevalence of *Salmonella* (3.1%) than other sampling locations, when *Salmonella* was present in piles the concentrations were high with 5 of the 10 (50.0%) positive samples found in piled litter containing ≥280,000 MPN/g (the upper limit of quantification; Table 2).

Prevalence findings from similar litter surveys conducted in the southeast have varied. One study by Lu et al. [23] collected litter and drag swabs directly from four houses while birds were present in Northeast Georgia; the litter had not been changed between flocks. No *Salmonella* were detected in litter using PCR targeting *invA* or from swab enrichment and subsequent streaking onto XLT-4 or Brilliant Green with Novobiocin. Another Georgia farm survey conducted by Martin et al. [24] failed to detect *Salmonella* in 86 poultry litter samples using the same pre-enrichment, enrichment, and selective agar protocols used in the current study. Conversely, in a study by Gu et al. [25] which was conducted in ten broiler houses at three farms in the Eastern Shore region of Virginia, litter (500g) was sampled monthly for *Salmonella* for a time ranging from 7 to 12 months (n = 102). Two composite 500 g samples were collected in each house and MPN enumeration included double strength lactose broth (pre-enrichment), tetrathionate broth (enrichment), and streaking onto XLT-4 plates. All ten broiler houses were positive for *Salmonella* at some point during the study with prevalence ranging from 14.3 to 35.4%. Finally, a North Florida survey of poultry litter found *Salmonella* in 61.1% of the 54 samples (30g each sample) collected from 18 farms [26]. The variability in detection among these studies could be attributed to multiple factors, including environmental conditions, rearing practices (e.g., flock vaccination or antibiotic use [27]), agricultural practices sampling or laboratory methodologies, litter age or treatment (e.g., composting [28]), and true variability of the pathogen prevalence in poultry litters. Regardless the cause, variability in prevalence across states, farms, and sampling locations indicates that risk mitigation strategies should account for potential contamination in all untreated litters.

Foodborne disease surveillance suggests that seasonality impacts infection prevalence, including infections caused by *Salmonella* [29, 30]. In soils and manures, warmer temperatures may facilitate survival. For instance, a survey of east Tennessee dairy farms found that *Salmonella* was more readily isolated from animal and environmental samples as ambient temperatures increased [31]. The current study was originally designed to examine seasonality, but farm participation and sample availability impacted when litter could be collected. Sampling month is indicated in Table 1, but too few samples were collected during the summer months to ascertain the role of seasonality. Additionally, the farms sampled were located in the southern region; this portion of the country has hot summers and mild winters, so had sufficient seasonality data been collected it may still have been difficult to determine the role of seasonality on *Salmonella* prevalence in litter. In fact, a 2020 survey of Florida poultry farms found no significant seasonal differences in *Salmonella* prevalence or concentration among samples collected during the spring, summer, and winter months [26].

Age of the sampled litter significantly (p<0.05) impacted *Salmonella* survival, and the current study found no *Salmonella* in poultry litter older than 21 days. This is in agreement with a study by Nicholson et al. that demonstrated that *Salmonella* in manure survives for less than one month in manure piles if temperatures reach 55°C [32]. It also may explain, in part, why *Salmonella* was not isolated in the previous Georgia survey conducted by Martin et al. [24] since most samples were older than 2 months. However, most of the samples (74.4%) in the Martin et al. work were also composted, which would also have impacted *Salmonella* levels in the material. Regardless, older litter (>21 days) appears to be at a lower risk for contamination as every day increase in age decreased the likelihood of isolating *Salmonella*. Produce growers could consider this when purchasing litter and request material that has been stored away from birds for at least a month instead of litter from a recently cleaned house.

The type of bedding material used for litter may impact *Salmonella* survival. The most common litter materials used in south Georgia, are sawdust, wood shavings, and peanut hulls [13]. Samples from the current study were most frequently comprised of wood shavings, sawdust, or straw. Other regions may use rice hulls, paper pulps, or sand for bedding material in poultry houses [13]. Collecting data on bedding material will be useful for future surveys to determine of *Salmonella* survival is impacted by bedding type. If some materials enhance survival, including after land application, fresh produce growers may be encouraged to avoid such litters.

The number of positive samples in the current work indicates that, despite previous studies indicating otherwise, *Salmonella* is a prevalent hazard within litter collected from poultry farms throughout the southern US. Further elucidating the intrinsic and extrinsic characteristics driving *Salmonella* survival and prevalence in litter is critical to mitigate potential microbial hazards when used as a soil amendment on fresh produce fields.

*Salmonella* serotype diversity varies by region. FoodNet surveillance in 2018 (the same year as the current study), indicated that the three most frequently identified serotypes causing illness in humans were Enteritidis, Newport, and Typhimurium [33]. Routine surveillance reported in 2016 by the US Department of Agriculture Food Safety and Inspection Service found that when broiler chicken carcasses were *Salmonella* positive, serotypes Kentucky, Enteritidis, and Typhimurium accounted for 60.8%, 13.6% and 7.7% of detected the serotypes, respectively [34, 35]. In the current study, Newport accounted for only 3% of isolates while Enteritidis and Typhimurium were not isolated. Newport has been the cause of numerous produce outbreaks in the U.S., including outbreaks linked to onions and cantaloupes [36]. While the outbreaks were not linked to the US southeast, both crops are important in this region, with onion and cantaloupe 2019 farmgate values of $133,179,945 and $12,915,395 in Georgia alone [37]. The production regions for these crops are also concentrated in areas with significant poultry production, so land application of litter to onion and cantaloupe fields frequently occurs [13]. The close proximity of these crops with the soil puts these commodities at particular risk for contamination by pathogens present in the soil.

Five of the six farms in the current study were positive at some point for more than one serotype, including some associated with U.S. produce outbreaks: Kiambu (imported papaya), Braenderup (shell eggs, mango), Mbandaka (breakfast cereal, tahini), and Saintpaul (cucumbers) [36]. The positive Texas farm had four distinct serotypes not found at any other farm. Serotype Michigan was only isolated from one Georgia farm and Saintpaul was only found at one Florida farm. Serotype Kentucky was the predominant serotype (46%) overall and was isolated from four of the six farms that were positive for *Salmonella*, making it the most widely distributed serotype (Table 3). Despite being one of the most frequently isolated serotypes isolated from poultry carcass rinses in the U.S. [34, 35], serotype Kentucky is less associated with human illness in the developed world [38]. Because of its increasing multi-drug resistance, including a high prevalence of ciprofloxacin resistance, some Kentucky strains (e.g., strain type 198) have been identified as emerging human pathogens in some parts of the world [38].

Many of the isolates from the current study have been identified in similar surveys in the region. The Gu et al. [25] broiler farm survey on the Eastern Shore of Virginia identified eight distinct serotypes out of 210 *Salmonella* isolates. The most frequently isolated serotypes were Typhimurium (64%), Kentucky (21%), and Thompson (7%). Like the current study which found that Newport comprised 3% of serotypes in southern poultry farms, 2% of the isolates in Virginia were Newport [25]. A 2020 litter survey of Florida farms isolated Typhimurium (27.7%), Kentucky (17.0%), Enteritidis (14.9%), and Mbandaka (14.9%; [26]).

During sample collection for the current project, what many poultry producers considered compost did not meet the FDA definition or treatment requirements to ensure microbial reduction [17]. Poultry farmers frequently described litter stored in dry stack sheds as compost, despite no monitoring or recording of times, pile temperatures or pile turnings. This distinction is important for growers of fresh produce to understand so they do not inadvertently apply untreated BSAAO to fresh produce while under the assumption the material has been composted.

Prevalence, distribution, concentration, and serotype appear to be highly variable, both within the current study and across studies conducted over the past several decades. Geographic location, litter type, bird age, bird type (i.e., broiler, breeder, layer), temperature, and many other factors can contribute to this disparity. Recommendations for the handling of litter should account for this variability and for other microbiological hazards, particularly if destined for land application to fields that will be used for or near fresh produce.

## Conclusion

*Salmonella* prevalence and concentration were highly variable throughout southern US farms, illustrating the importance of proper composting to reduce food safety risks from *Salmonella* and other pathogens found in poultry manure [10]. Sample age and location significantly impacted *Salmonella* prevalence, underscoring the importance of considering litter storage conditions and the interval between last bird contact and application to production land when assessing microbial hazards. This study provides valuable information regarding variability in microbiological quality of poultry litter and factors that may reduce the microbial load, consequently minimizing the risks associated with the use of BSAAO on fresh produce fields.

## Supporting information

S1 Table *Salmonella* prevalence and concentration data in poultry litter collected from southern U.S. farms. (XLSX)
